# Refractory dermatomyositis responsive to anifrolumab

**DOI:** 10.1016/j.jdcr.2023.10.023

**Published:** 2023-11-07

**Authors:** Phillip S. Ang, Ekene Ezenwa, Kichul Ko, Mark D. Hoffman

**Affiliations:** a Pritzker School of Medicine, The University of Chicago, Chicago, Illinois; b Section of Dermatology, Department of Medicine, The University of Chicago Medical Center, Chicago, Illinois; c Section of Rheumatology, Department of Medicine, The University of Chicago Medical Center, Chicago, Illinois

**Keywords:** anifrolumab, antibody, biologic, dermatomyositis, interferon

## Introduction

Dermatomyositis (DM) is a connective tissue disorder with dermatologic and/or extracutaneous manifestations. Although DM primarily affects skin and muscle, the disease can cause pathologic changes to other organs such as the lungs, and may be associated with malignancy. Various treatments are deployed in DM management, but their effects are inconsistent. Skin disease can be refractory to therapy, even when other involved organ systems are responsive.^1^ Interferons (IFNs) are believed to play a role in driving DM disease activity, and medications targeting IFN pathways are both available and under development. In this report, we present an individual with DM whose refractory skin disease responded to anifrolumab, a monoclonal antibody blocking IFN-α receptor-1.

## Case report

**Fig 1:**
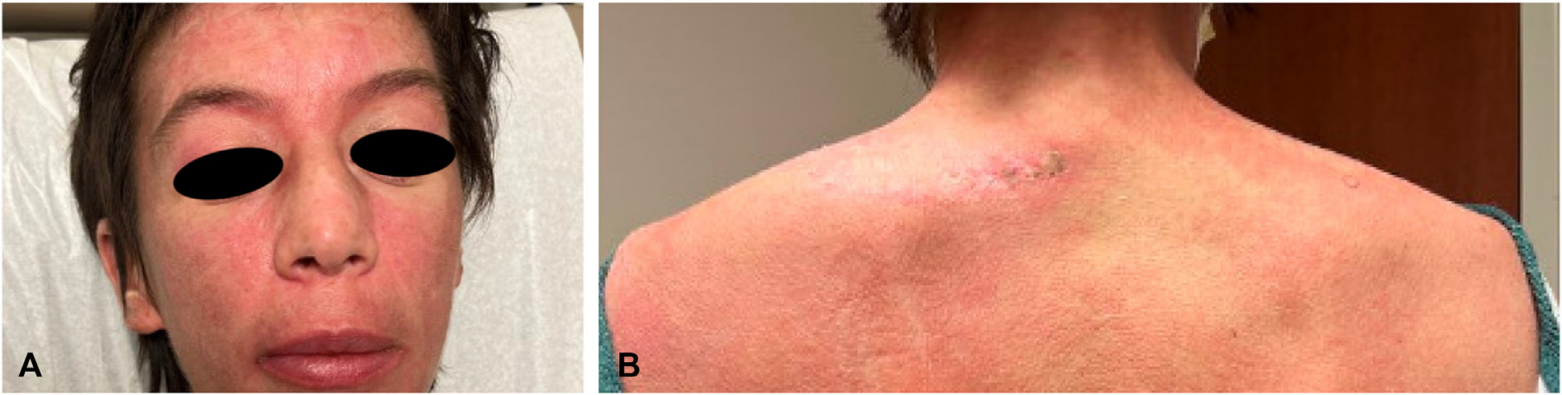
Dermatomyositis of **(A)** the face and **(B)** upper portion of the back at presentation.

A 43-year-old woman with a history of metastatic breast cancer and pruritic rashes of the face, hands, and torso ongoing for a few years presented with worsening skin disease, including newer involvement of the eyelids and scalp. There was now associated fatigue, proximal muscle weakness, and joint stiffness. Ductal breast carcinoma had been diagnosed 2 years earlier—with lung and lymph node metastases occurring a year later—which had been treated with chemotherapy and radiation therapy, resulting in largely stable nonprogressive disease that was being managed palliatively. On examination, there were violaceous patches and plaques typical of DM: on the face, including a heliotrope discoloration of the eyelids; on the fingers, with Gottron papules; and characteristic lesions on the chest, back, lateral aspect of the hips and thighs (Fig 1, *A* and *B*). Ragged cuticles and periungual telangiectasias were also present. Punch biopsies of the dorsal aspect of the hand and upper portion of the back demonstrated a vacuolar dermatitis consistent with DM. Creatine kinase was mildly elevated at 202 U/L (ref 9-185); myositis antibody testing was positive for transcriptional intermediary factor 1-gamma at 34 U (ref <20 U) (Labcorp). Magnetic resonance imaging demonstrated mild edema-like signals of thigh muscles compatible with myositis.

**Fig 2:**
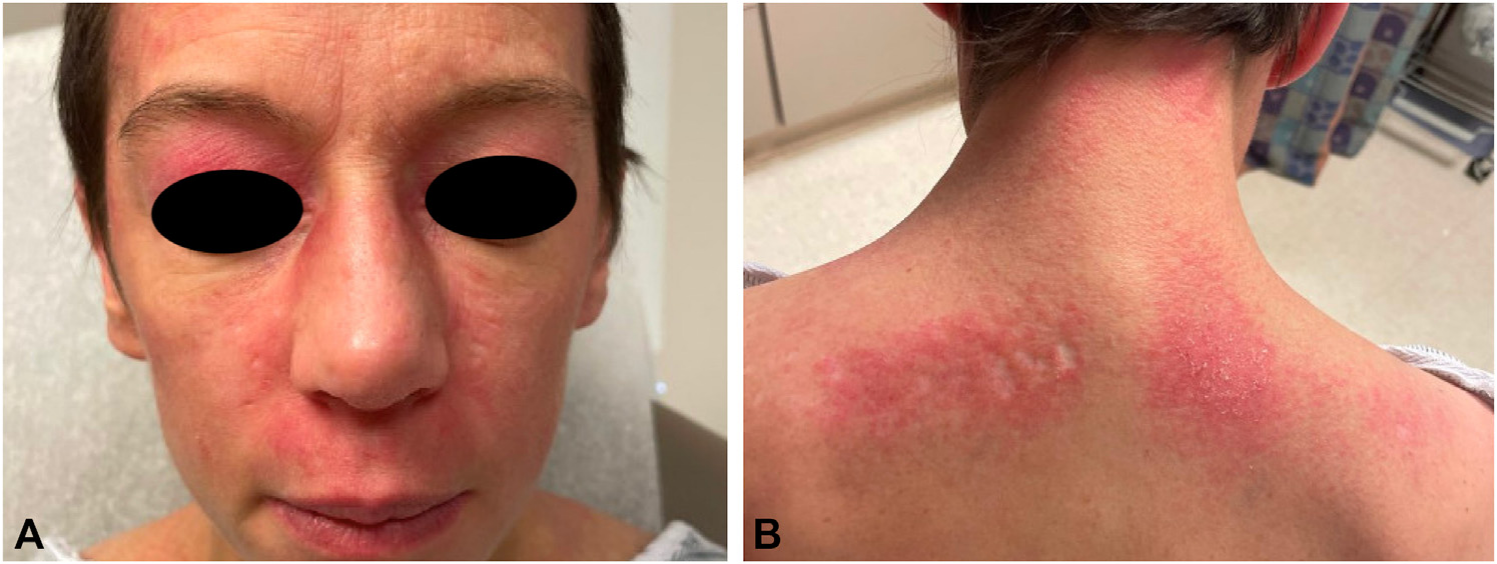
**(A)** Face and **(B)** upper portion of the back after 5 cycles of intravenous immunoglobulin, and before anifrolumab administration.

**Fig 3:**
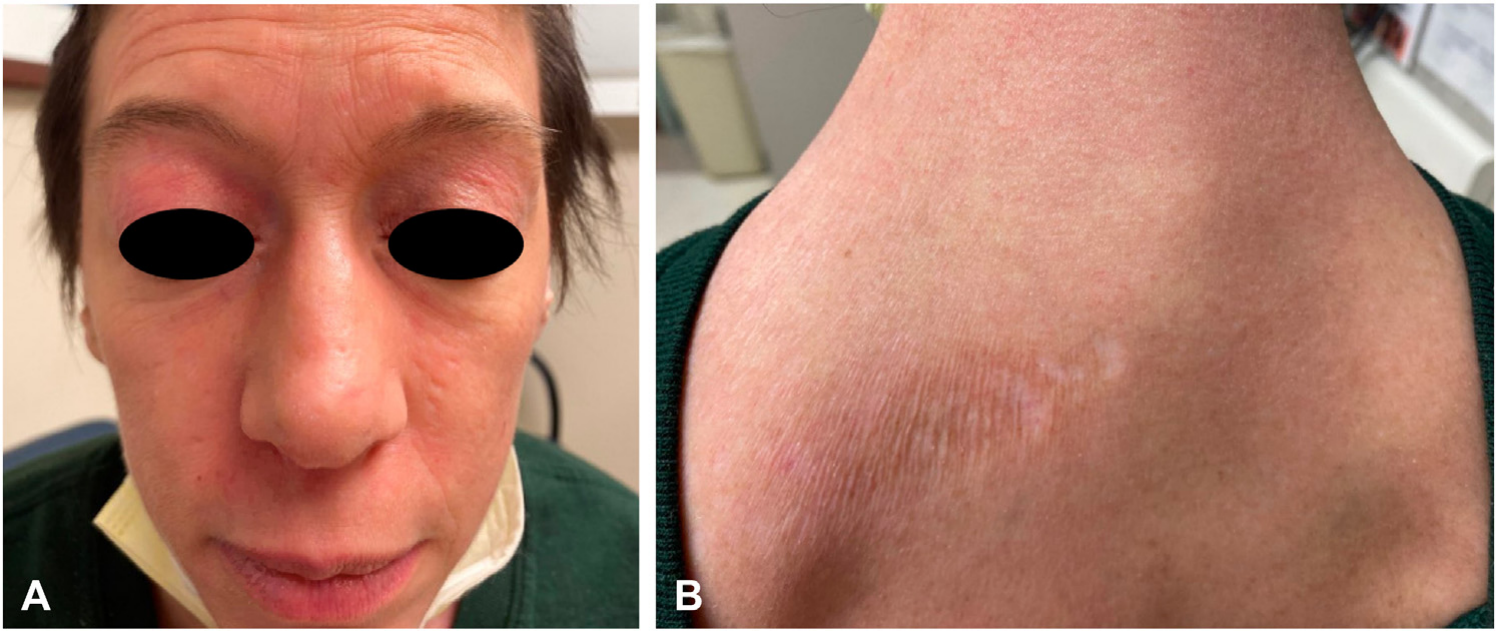
Improvement of **(A)** the face and **(B)** back following 1 dose of anifrolumab.

**Fig 4:**
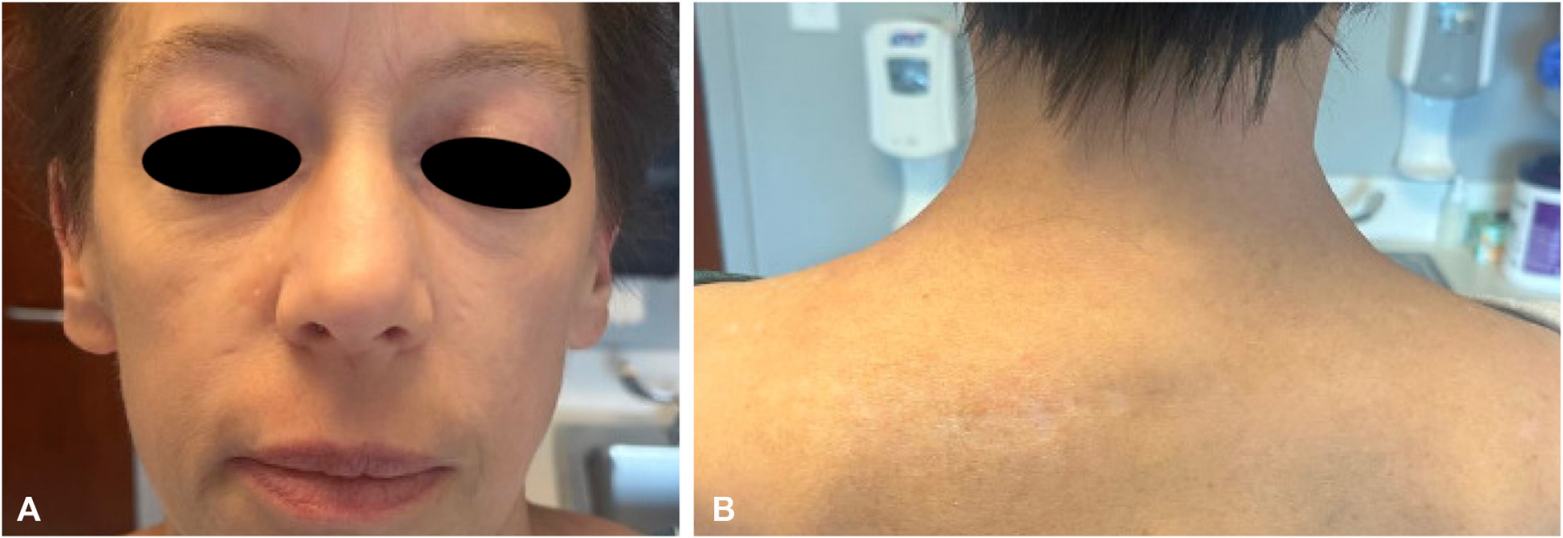
Resolution of **(A)** facial and **(B)** back activity following 4 doses of anifrolumab.

Treatment was commenced with hydroxychloroquine 200 mg daily (approximately 5 mg/kg/day), but after 2 weeks, she developed a morbilliform (presumably drug-) eruption of the torso and extremities leading to its discontinuation. Topical agents to include corticosteroids, tacrolimus, and ruxolitinib had negligible effects. Prednisone was begun and dosed at 1 mg/kg/day, which lessened the skin manifestations, but cutaneous signs and symptoms still remained severe and extracutaneous disease was progressive. Intravenous immunoglobulin dosed at 2 g/kg monthly was initiated, and after 5 cycles muscle symptoms had improved, but there was little change in the skin lesions and only modest improvement in the pruritus (Fig 2, *A* and *B*). Anifrolumab 300 mg monthly was then begun (and intravenous immunoglobulin discontinued), chosen based on its rapid effect when used for cutaneous lupus,^2^ and its promising safety data—including malignancy rate comparable to placebo—as described in a long-term extension safety study.^3^ Anifrolumab administration resulted in marked skin improvement of the torso that was apparent after the first injection (Fig 3, *A* and *B*), and was continued for an additional 3 doses, leading to near-clearing of the facial activity. At that time, progressive metastatic hepatic, osseous, and lymph node disease was identified, chemotherapy was then reinstituted, and the decision was made to pause treatment with anifrolumab. Skin disease remained under excellent control for 2 months after the last anifrolumab injection (Fig 4, *A* and *B*), at which point the face, scalp, neck, and chest flared, prompting plans to resume the anifrolumab.

## Discussion

Treatments for DM vary and can include topical, oral, and intravenous medications. The selection of specific agents depends on an individual patient’s disease activity, comorbidities, and risk tolerance.^4^^,^^5^

Newer targeted therapies continue to develop as more is discovered about DM-associated inflammatory pathways. IFNs are a family of cytokines theorized to play a role in driving DM disease activity. To date, there are 3 known groups of human IFNs: type I including numerous IFN-αs as well as IFN-β, IFN-ε, IFN-κ, and IFN-ω, all which bind to the same receptor complex having IFN-αR1 (aka IFNAR1) and IFNAR2 components; type II, whose single member is IFN-γ; and type III, comprising several IFN-λs.^6^ Serologic and gene expression studies have found a positive correlation between levels of IFN-β and DM disease and its activity.^7^^,^^8^ These findings collectively support the notion that aberrant IFN-β signaling is involved in the pathophysiology of DM.

We report an individual with DM whose intravenous immunoglobulin-refractory skin disease responded to the IFNAR1-blocking antibody anifrolumab. Anifrolumab was first approved in 2021 for the treatment of moderate to severe systemic lupus erythematosus.^9^ Systemic lupus erythematosus and DM share several common features including their involvement of the skin and potential for extracutaneous manifestations, and interestingly, both diseases have been linked to dysregulation primarily assigned to type I IFN pathways.^8^ In contrast to DM and the role imputed to IFN-β, the pathophysiology of systemic lupus erythematosus is more closely tied to IFN-α.^10^ Although different IFNs have been implicated in these 2 diseases, they act through a common receptor. Accordingly, anifrolumab may have applications in the management of recalcitrant DM, as described herein.

## Conflicts of interest

None disclosed.
